# Genotyping tools and resources to assess peanut germplasm: smut-resistant landraces as a case study

**DOI:** 10.7717/peerj.10581

**Published:** 2021-01-29

**Authors:** Alicia N. Massa, Marina Bressano, Juan H. Soave, Mario I. Buteler, Guillermo Seijo, Victor S. Sobolev, Valerie A. Orner, Claudio Oddino, Sara J. Soave, Paola C. Faustinelli, Francisco J. de Blas, Marshall C. Lamb, Renee S. Arias

**Affiliations:** 1 National Peanut Research Laboratory, USDA-ARS, Dawson, GA, USA; 2 Facultad de Ciencias Agropecuarias, Universidad Nacional de Córdoba, Córdoba, Argentina; 3 Criadero El Carmen, General Cabrera, Córdoba, Argentina; 4 Instituto de Botánica del Nordeste (IBONE, CONICET-UNNE) and Facultad de Ciencias Exactas y Naturales y Agrimensura, Universidad Nacional del Nordeste, Corrientes, Argentina; 5 Instituto Multidisciplinario de Biología Vegetal-(IMBIV-CONICET-UNC), Córdoba, Argentina

**Keywords:** Peanut Smut, *Arachis hypogaea*, *Thecaphora frezii*, Peanut, SNP genotyping, rhAmp assay, Genetic introgression

## Abstract

Peanut smut caused by *Thecaphora frezii* is a severe fungal disease currently endemic to Argentina and Brazil. The identification of smut resistant germplasm is crucial in view of the potential risk of a global spread. In a recent study, we reported new sources of smut resistance and demonstrated its introgression into elite peanut cultivars. Here, we revisited one of these sources (line I0322) to verify its presence in the U.S. peanut germplasm collection and to identify single nucleotide polymorphisms (SNPs) potentially associated with resistance. Five accessions of *Arachis hypogaea* subsp. *fastigiata* from the U.S. peanut collection, along with the resistant source and derived inbred lines were genotyped with a 48K SNP peanut array. A recently developed SNP genotyping platform called RNase H2 enzyme-based amplification (rhAmp) was further applied to validate selected SNPs in a larger number of individuals per accession. More than 14,000 SNPs and nine rhAmp assays confirmed the presence of a germplasm in the U.S. peanut collection that is 98.6% identical (*P* < 0.01, bootstrap *t*-test) to the resistant line I0322. We report this germplasm with accompanying genetic information, genotyping data, and diagnostic SNP markers.

## Introduction

Peanut smut caused by the fungus *Thecaphora frezii* Carranza & Lindquist is a severe soil borne disease endemic to Argentina and Brazil (Rago et al., 2017). It was first described in a wild diploid peanut germplasm collected in Aquidauana, Mato Grosso do Sul, Brazil (Carranza & Lindquist, 1962), later identified as *Arachis kuhlmannii* Krapov. & W.C. Gregory nov. sp. (Krapovickas & Gregory, 1994). Currently, the pathogen has been observed in 100% of the peanut production area of Argentina, where disease incidence up to 52% and yield losses of 35% has been reported (Paredes et al., 2016; Rago et al., 2017).

It is imperative to identify sources of smut-resistance in peanut germplasm collections. However, a safe screening for resistance remains limited to its endemic areas (Arias et al., 2019; Kingsolver, Melching & Bromfield, 1983; Rago et al., 2017). Extensive screening in Argentina, which included wild diploid species of *Arachis* (2*n* = 2*x* = 20), landraces, advanced breeding lines, and elite peanut varieties, led to the identification of resistant germplasm (Bressano et al., 2019; de Blas et al., 2019). Wild diploid species with resistance to smut were used in interspecific crosses to develop pre-breeding materials for introgression (de Blas et al., 2019). Bressano et al. (2019) reported two smut resistant landraces of the tetraploid *Arachis hypogaea* L. (2*n* = 4*x* = 40), then used simple-sequence repeats and Insertion/Deletion markers to confirm its introgression into elite peanut cultivars. These landraces were originally collected from South America as part of international efforts that involved several expeditions to the peanut center of origin (Barkley et al., 2016; Simpson & Higgins, 1984; Simpson et al., 1992; Valls et al., 1985) and added to the world germplasm collection, thus making this germplasm accessible to peanut researchers and breeders. The resistant germplasm of Bressano et al. (2019), however, has not been verified in the U.S. germplasm collection. With high-density single nucleotide polymorphism (SNP) data, this valuable germplasm could be traced back to landraces introduced from South America.

The U.S. peanut germplasm collection held at the USDA Plant Genetic Resources Conservation Unit in Griffin, Georgia, is a valuable source of genetic diversity. The collection consists of 9,321 accessions of cultivated peanut (*A. hypogaea*) and 655 accessions from 66 wild *Arachis* species (Barkley et al., 2016). Accessions from South America, particularly landraces from Bolivia (Krapovickas et al., 2009; Simpson & Higgins, 1984) as those used in the present study, were often collected as phenotypically heterogeneous mixtures, and despite the efforts to characterize this germplasm (Chen et al., 2014; Otyama et al., 2019), there is still limited information regarding the genetic variation between and within accessions.

The recent development of the 48K SNP peanut array (Axiom_Arachis2) has provided a set of single-nucleotide polymorphism markers that can be used to examine genome-wide patterns of genetic variation in diploid and tetraploid *Arachis* germplasm (Clevenger et al., 2017, 2018). The array was designed to include sequence polymorphism from both diploid (*A. batizocoi*, *A. cardenasii*, *A. duranensis*, *A. magna*, *A. stenosperma*) and tetraploid (*A. hypogaea* subspecies *hypogaea* and *fastigiata*) species (Clevenger et al., 2018).

The objectives of the present study were to: (1) verify whether the source of a smut resistant line used by Bressano et al. (2019) was present in the U.S. peanut germplasm collection, and (2) provide genomic tools and germplasm resources to help develop smut resistant breeding lines in the U.S. without the risk of introducing the pathogen. The study further re-examined the 16 recombinant inbred lines (RILs) from Bressano et al. (2019) population (JS31411) to assess potential associations between SNP markers and smut resistance. Large-scale and small-scale genotyping platforms were combined to genotype five Plant Introduction (PI) accessions of *A. hypogaea* subsp. *fastigiata*, the smut resistant line I0322, and the 16 RILs (Bressano et al., 2019). A PCR allelic discrimination platform called rhAmp SNP genotyping (Beltz et al., 2018; Dobosy et al., 2011) was further applied to validate selected SNPs in a larger number of individuals per accession. We report the U.S. germplasm source of a smut resistant line with accompanying genotyping data and diagnostic SNP markers.

## Materials and Methods

### Plant material

This study utilized two sources of plant DNA: (1) five accessions of *A. hypogaea* subsp. *fastigiata* var. *fastigiata* obtained from the U.S. Department of Agriculture, Agricultural Research Service, National Plant Germplasm System (NPGS), Plant Genetic Resources Conservation Unit peanut (*Arachis*) collection in Griffin, GA, PI 497287, PI 497283, PI 497284, PI 497285, and PI 497286 (hereinafter referred to as NPRL-S118 to NPRL-S122, respectively); and (2) a smut resistant line I0322, along with 16 RILs (resistant *F*_**2:5-7**_ lines) derived from the cross I0322 × Guasu (JS31411), provided by the Criadero El Carmen, General Cabrera, Córdoba, Argentina (Bressano et al., 2019). I0322 was originally selected from a landrace of *A. hypogaea* subsp. *fastigiata* collected in Bolivia as part of the *Arachis* germplasm collections conducted in South America between 1976 and 1983 (Simpson & Higgins, 1984). This germplasm was introduced into the NPGS under a unique U.S. name and further assigned to five PI numbers (Simpson et al., 1992), all of which were included in this study to verify the genetic identity of I0322.

### SNP genotyping using the Axiom Arachis2 SNP array

Two plants per PI accession were grown in the greenhouse at the USDA-ARS National Peanut Research Laboratory (NPRL) in Dawson, GA, and young leaves from individual plants were used for DNA extraction. DNA was extracted from freeze-dried leaves using the QIAGEN DNeasy Plant Mini Kit (QIAGEN, Germantown, MD, USA), quantified by spectrophotometry (NanoDrop 2000, Thermo Fisher Scientific, Waltham, MA, USA), and adjusted to a concentration of 30 ng/μL. DNA from I0322 and 16 RILs was provided by Criadero El Carmen (Bressano et al., 2019). All samples were genotyped with the Affymetrix Axiom Arachis2 48K SNP array (Clevenger et al., 2018). Marker quality assessment and SNP calling was conducted using the Axiom Analysis Suite v5.0.1 software, following the Axiom best practices genotyping workflow with option set to *polyploid* (Thermo Fisher Scientific Inc., Waltham, MA, USA). All samples with a dish quality control (dQC) value ≥ 0.82 and QC call rate ≥ 0.97 were considered to have passed the first quality control. A more stringent metric threshold was further applied to assign SNPs to the PolyHighResolution (PHR) category using a Fisher’s Linear Discriminant (FLD) ≥ 5.6 and a FLD for the homozygous genotype cluster (HomFLD) ≥ 10 (the default for polyploids is ≥ 3.6). Genotype data were extracted only from the PHR conversion type. Additional filtering steps were performed to remove SNPs called as heterozygous, having > 10% missing genotype calls, or showing divergence between technical replicates. The genome location of SNPs was based on the Axiom Arachis2 SNP array, as described in the Table S1 of Clevenger et al. (2018). Targeted SNPs were also mapped to the *A. hypogaea* cv. Tifrunner reference genome (https://peanutbase.org).

### Germplasm identity and accession relationships

To help understand genetic relationships between genotypes and to explore the probability of identity, that is, the probability that two alleles, one from individual *x* and one from individual *y* are identical in state, we used the measure of allele sharing distances (ASDs) (Gao & Martin, 2009; Gao & Starmer, 2007). The distance matrix was calculated as one minus the proportion of shared alleles between pairs of individuals using the *propShared* function of the *adegenet* package (Jombart, 2008) in R version 3.6.1 (R Development Core Team, 2019). One of the advantages of using ASDs is that no allele frequency information is required, which makes this method valid when the sample size is small, as is the case here. Furthermore, with a large number of genome-wide SNP loci, ASDs provide adequate information for use in genetic relationship inference (Gao & Starmer, 2007). The significance of the observed differences in the ASDs was determined with a bootstrap *t*-test statistic with 1,000 repetitions.

Genetic relationships among accessions were further assessed in a broader context using an existing array-based SNP dataset consisting of 20 genotypes (Table S2 in Clevenger et al., 2018). Only PHR SNPs that were common to both datasets were considered. A neighbor-joining analysis using ASDs, and a principal component analysis (PCA) were performed to identify genetically related genotypes. All statistical analyses were conducted utilizing the *bionj* function in *ape* and the *glPca* in *adegenet* packages as implemented in R Development Core Team (2019).

### RNase H2-dependent PCR genotyping assays

A dual enzyme chemistry technology called rhAmp SNP genotyping (Beltz et al., 2018) (Integrated DNA Technologies, Skokie, IL, USA) was used to validate a set of selected array-based SNPs in a larger number of individuals per accession. This novel technology called rhAmp is based on the RNase H2-dependent polymerase chain reaction (rhPCR) and universal reporters (Dobosy et al., 2011). Candidate SNPs for the rhAmp assays were randomly selected from a set of true polymorphic SNPs identified with the Axiom Arachis2 array. The selected SNPs with corresponding flanking sequences (from the Axiom Arachis2 SNP array) were submitted to the rhAmp Genotyping Design Tool at IDT (Integrated DNA Technologies, Skokie, IL, USA) and based on the strength of the thermodynamics, the highest ranked assays were retained. To meet the technical requirements for primer design, flanking sequences shorter than 50 bp were extended up to 50–60 bp (on either side) based on the *A. hypogaea* reference genome sequence at the probe target sites (https://peanutbase.org). For each assay, rhAmp utilizes two allele specific primers and a locus specific primer (Table S1). To confirm primer specificity, the resulting rhAmp SNP assay primers were subjected to BLAST analysis against the reference genomes of the tetraploid *A. hypogaea* (Bertioli et al., 2019) and diploids *A. duranensis* and *A. ipaënsis* (Bertioli et al., 2016).

Polymerase chain reaction amplification reactions were prepared according to the protocol for genotyping with rhAmp SNP assays using rhAmp genotyping master mix and rhAmp reporter mix with reference dye (Integrated DNA Technologies, Skokie, IL, USA). Fifteen ng of sample DNA were used in 10 µL reactions with the following parameters: a pre reading stage 30 s 60 °C; enzyme activation 10 min 95 °C; then 40 cycles of: 10 s 95 °C, 30 s 60 °C, 20 s 68 °C; and a final post reading stage of 30 s at 60 °C. Thermal cycling was performed in a QuantStudio 7 Flex Real-Time PCR System (Applied Biosystems; Thermo Fisher Scientific, Waltham, MA, USA). Each accession was represented by five biological replicates, which included the original DNA samples used on the array as a reference. The total number of rhAmp assays was (9 SNPs × 5 genotypes × 5 biological replicates × 2 technical replicates) + (9 SNPs × 1 genotype (I0322) × 2 technical replicates) + 80 no-template controls (NTC) = 548. Analyses were performed with the QuantStudio Real-Time PCR Software v1.3 (Thermo Fisher Scientific, Waltham, MA, USA) using the general workflow for genotyping experiments.

### Marker-trait association

Given the small number of recombinant inbred lines available for analysis, association between disease resistance and SNP markers was conducted by running simple linear regression followed by multiple linear regression (using forward stepwise regression). The analysis used only SNPs that were polymorphic between the parental lines (I0322 and Guasu) and segregated in a 1:1 ratio. Marker deviation from the expected ratio was determined by a chi-square test of goodness of fit, and those SNPs with a *P* < 0.01 were removed. The coefficient of determination (adjusted *R*^2^) was used to measure the proportion of phenotypic variation explained by the SNP marker (Collard et al., 2005). For those SNPs with significant adjusted *R*^2^ (*P* < 0.05), the analysis of variance and Tukey test were applied to determine allele effects. Disease incidence (IN) and disease severity index (DI) mean values were extracted from Table 2 and Table S1 of Bressano et al. (2019). An additional RIL, I-3, with a DI of 0.032 and a IN of 4.5, was added to the present study. All IN and DI mean values are listed in Table S2. Disease incidence and DI were calculated using equations (1) and (2) of Bressano et al. (2019). The severity classes used in the calculation of DI were determined based on a 0–4 scale, where 0 = healthy pods; 1 = normal pod with a small sorus in single kernel; 2 = deformed or normal pod with half of the kernels affected; 3 = deformed pod and one completely smutted kernel; and 4 = deformed pod with all kernels completely smutted (Rago et al., 2017). All statistical analyses were performed in R using the *lm*, *aov*, and *LTukey* functions (R Development Core Team, 2019). The extent of linkage disequilibrium (*r*^2^) and haplotype structure at significant SNP loci was estimated and visualized with Haploview version 4.2 (Barrett et al., 2004).

## Results

### SNP genotyping with the Axiom Arachis2 SNP array

Out of the 47,837 SNPs available from the 48K Axiom Arachis2 SNP array (Clevenger et al., 2018), 14,837 SNPs were successfully extracted from the PHR conversion type. After quality filtering to remove SNPs with heterozygous genotype calls and SNPs with > 10% missing values (2.7%), 14,298 SNPs were available for downstream analyses (Table S3). SNPs were distributed across the 20 chromosomes, providing whole-genome coverage with an average of 714 SNPs per chromosome (Table S4). Some SNPs (26) were on scaffolds, not anchored to the sequences of the reference genomes.

The PI accessions and the resistant line (I0322) were highly homozygous with an overall number of 137 heterozygous calls. Most of these heterozygous calls were dispersed in the genome, which are most likely genotyping errors. However, a group of 45 SNPs occurred in continuous blocks, mapped to the distal end of homoeologous group 4 (Arahy.04/Arahy.14) of *A. hypogaea* cv. Tifrunner (Table S5). This region of cv. Tifrunner has undergone homoeologous recombination and presents a tetrasomic genome conformation (BBBB) with segments of the B genome transferred into the A genome (Bertioli et al., 2019; Clevenger et al., 2018; Clevenger et al., 2017). BLAST search of probe sequences against the genome of cv. Tifrunner indicated no allelic differentiation between the two sub-genomes at the 45 SNP loci (https://peanutbase.org). In this study, four of the PI accessions (NPRL-S118–NPRL-S121) and the resistant line exhibited heterozygous calls at all 45 loci, while accession NPRL-S122 was homozygous. The high sequence similarity between sub-genomes suggest that most heterozygous calls in this region are homoeologous SNPs. Although, some heterozygous calls may also be indicative of paralogous SNPs due to duplicated gene copies. This region is enriched for disease resistance and defense response genes of the Toll/Interleukin-1 Receptor (TIR)-Nucleotide Binding Site (NBS)-Leucine-Rich Repeat (LRR), Chitinase, F-box, and WD repeat-containing proteins families (Table S5).

### Genotype verification and accession relationships

Genetic distances (based on ASDs) ranged from 0.014 (between NPRL-118 and I0322) to 0.271 (between NPRL-S120 and NPRL-S122). In terms of the proportion of shared alleles, these values translated into 0.986 and 0.729, respectively (Table 1). Among the five PI accessions, NPRL-S118 was the closest (0.986) to the resistant line I0322, separated by only 195 (< 1.4%) SNP differences; while accession NPRL-S122 was the most distant with up to 3,785 (26.5%) SNP differences (Table 1). The neighbor-joining tree of genetic distances defined a group of four closely related PI accessions (NPRL-S118, NPRL-S119, NPRL-S120, NPRL-S121) and a distant single cluster with accession NPRL-S122 (Fig. S1). The resistant line I0322 clustered together with accession NPRL-S118, which was anticipated based on the ASD values and seed testa color (Fig. S1). However, none of the four closely related PI accessions were significantly different from I0322 (*P* > 0.01, bootstrap *t*-test). In contrast, ASD values for all pairwise comparisons that included accession NPRL-S122 were statistically highly significant (*P* < 0.001, bootstrap *t*-test) (Table 1). Morphologically, accession NPRL-S122 presents different pod reticulation and pod shape (Fig. S1), which is described as “peruviana” type (https://npgsweb.ars-grin.gov/gringlobal/). Thus, it is likely that NPRL-S122 is one of the landraces of *A. hypogaea* subsp. *fastigiata* var. *peruviana* documented for Bolivia (Krapovickas et al., 2009), which was collected together with the *fastigiata* type.

**Table 1:** Allele sharing distance (below diagonal) and proportion of shared alleles (above diagonal) between pair of genotypes, based on 14,298 SNPs.

Sample ID	NPRL-S119	NPRL-S120	NPRL-S121	NPRL-S122	NPRL-S118	I0322
NPRL-S119	–	0.970	0.957	0.739	0.955	0.948
NPRL-S120	0.030	–	0.963	0.729	0.974	0.966
NPRL-S121	0.043	0.037	–	0.735	0.971	0.971
NPRL-S122	0.263***	0.271***	0.265	–	0.734	0.735
NPRL-S118	0.045	0.026	0.029	0.266***	–	0.986
I0322	0.052	0.034	0.029	0.265***	0.014	–

**Note:**

*** Statistically significant values (*P* < 0.001, bootstrap *t*-test).

To help understand the genetic relationships between the PI accessions and the resistant line I0322, the distribution of the number of SNP differences between each accession and I0322 was plotted along the 20 chromosomes (Fig. S2). The frequency distribution of SNP differences reflected the observed accession relationships, and except for accession NPRL-S122, these differences occurred more frequently towards the ends of the chromosome arms where relative gene density and recombination frequency is generally increased (Fig. S2) (Nagy et al., 2012; Ren et al., 2018).

**Figure 1:**
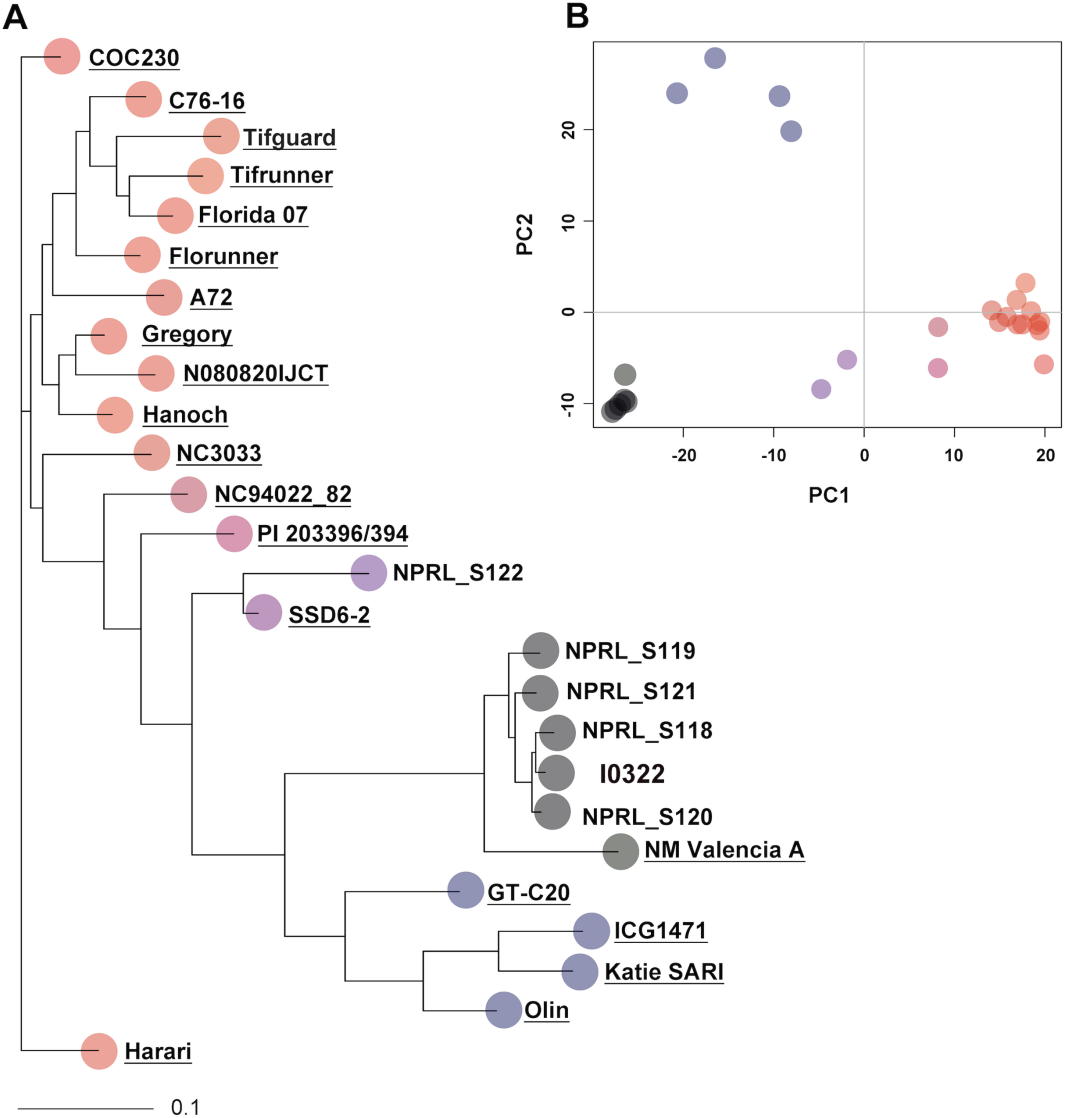
Neighbor-joining (NJ) tree and principal component analysis (PCA) of 26 genotypes, constructed with 4,871 SNPs. (A) The NJ tree is based on allele share distances. The underlined names are the 20 genotypes extracted from Clevenger et al. (2018). (B) The PCA plot shows the projection of genotypes on the two-dimensional space spanned by the first two principal components. Genotypes in the PCA plot are represented with the same color as in the NJ tree.

To assess genotype relationships in a broader context, the five PI accessions and the resistant line were compared with 20 peanut genotypes from Clevenger et al. (2018), using a total of 4,871 SNPs (indicated with asterisks in Table S3). Pairwise genetic distances ranged from 0.013 to 0.563 with 56 and 2,774 SNP differences, respectively (Table S6). As indicated by cluster analysis and PCA, accession NPRL-S118 remained the closest genotype to the resistant line I0322, and together with accessions NPRL-S119, NPRL-S120, and NPRL-S121, clustered with cultivar NM Valencia-A (*A. hypogaea* L. subsp. *fastigiata*) (Fig. 1). Nevertheless, NM Valencia-A was significantly different (*P* < 0.001, bootstrap *t*-test) from the five genotypes in the cluster.

### SNP genotyping using rhAmp assays

**Figure 2:**
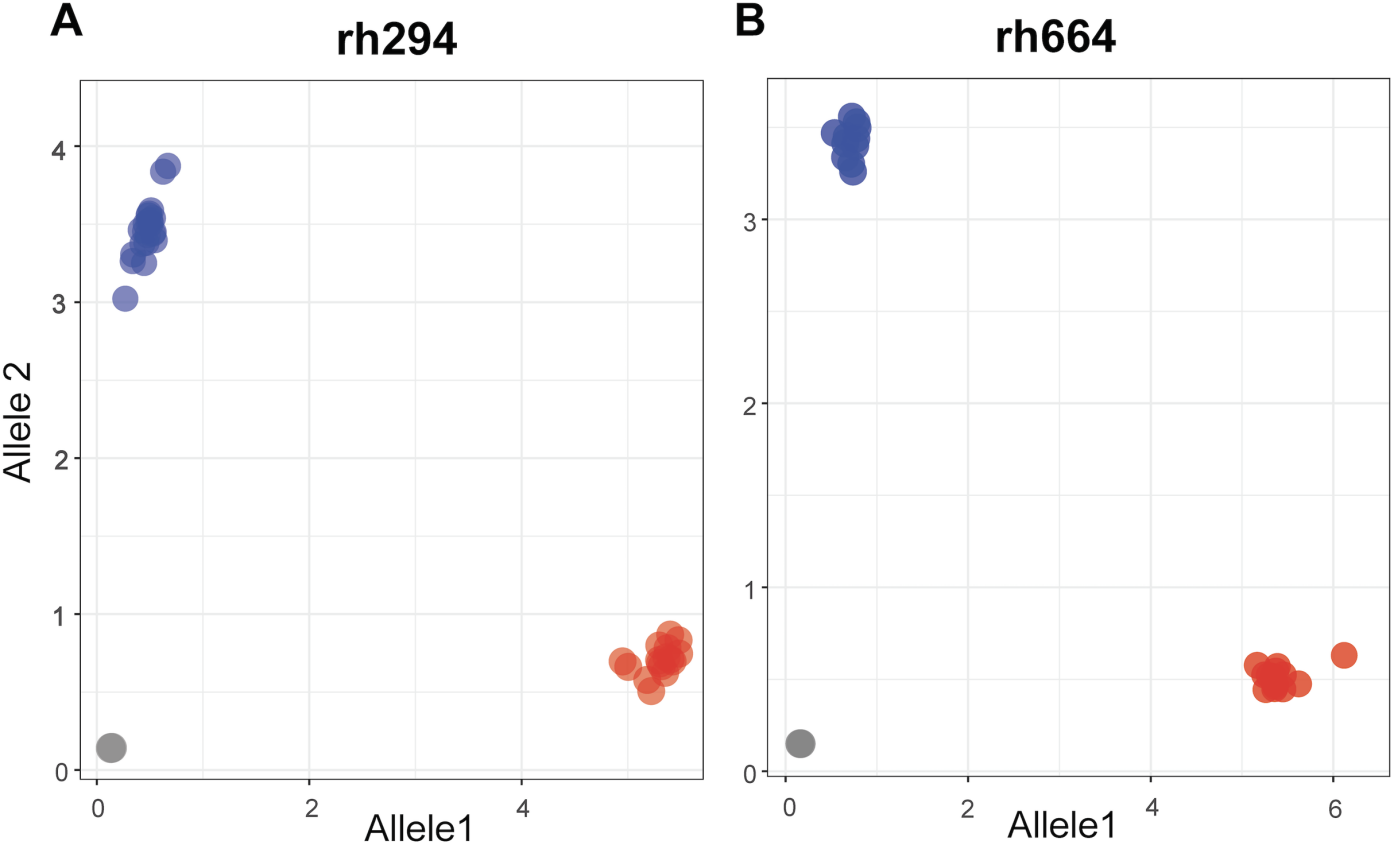
Allelic discrimination plots showing the rhAmp genotyping assays rh294 (A) and rh664 (B) using 52 samples, 26 genotypes in duplicate. Blue and red dots represent the homozygous genotypes for allele 1 (C/C or T/T) and allele 2 (A/A or C/C), respectively. The gray dots on the bottom left of the plot represent the no-template control.

Single nucleotide polymorphisms for the rhAmp assays were selected from a set of 4,247 SNPs showing polymorphism in at least one pairwise comparison. A final set of nine rhAmp assays was chosen to genotype 52 DNA samples (26 biological samples × 2 technical replicates). The assays targeted unlinked SNP loci from nine chromosome regions of the peanut genome (Table S1). The call-rate, defined as the percentage of genotype calls relative to the total number of genotypes, was 100% (Table 2), with unambiguous allelic discrimination between genotypes, and SNP calls confirmed by technical replicates. Examples of allelic discrimination for assays rh294 and rh664 are provided in Figs. 2A and 2B. Ninety percent of the genotype calls was as expected based on the Axiom Arachis2 SNP array, only four were unexpected, one in assay rh621, one in assay rh506, and two in assay rh921. Heterozygous instead of homozygous calls were observed in both cases, suggesting the occurrence of genome-specific nucleotide differences in the target sequence of the array-based SNP, not covered by the rhAmp primer sequence (Table 2). Therefore, a heterozygous call is more likely to be the result of genome-specific alleles (homoeologous SNPs) rather than true heterozygosity.

**Table 2:** Genotype scores of the Axiom Arachis2 SNP array markers in five PI accessions (five biological replicates) and the resistant line I0322, based on nine rhAmp assays.

Genotype	Biological replicate	rhAmp assay ID with SNP alleles								
		rh621 (C/T)	rh664 (A/C)	rh019 (A/G)	rh147 (A/C)	rh294 (C/T)	rh921 (A/G)	rh506 (A/G)	rh688 (A/G)	rh194 (A/C)
NPRL-S119	1	T/T	A/A	G/G	C/C	C/C	A/G	G/G	A/A	C/C
	2	T/T	A/A	G/G	C/C	C/C	A/G	G/G	A/A	C/C
	3	T/T	A/A	G/G	C/C	C/C	A/G	G/G	A/A	C/C
	4	T/T	A/A	G/G	C/C	C/C	A/G	G/G	A/A	C/C
	5	T/T	A/A	G/G	C/C	C/C	A/G	G/G	A/A	C/C
NPRL-S120	1	C/T	A/A	G/G	A/A	T/T	A/A	A/A	G/G	A/A
	2	T/T	A/A	G/G	A/A	C/C	A/A	G/G	A/A	A/A
	3	T/T	A/A	G/G	A/A	C/C	A/A	G/G	A/A	A/A
	4	C/T	A/A	G/G	A/A	T/T	A/A	A/A	G/G	A/A
	5	C/T	A/A	G/G	A/A	T/T	A/A	A/A	G/G	A/A
NPRL-S121	1	T/T	A/A	A/A	A/A	T/T	A/A	G/G	G/G	C/C
	2	T/T	A/A	A/A	A/A	T/T	A/A	G/G	G/G	C/C
	3	T/T	A/A	A/G	A/A	T/T	A/A	A/G	A/G	A/C
	4	T/T	A/A	A/A	A/A	T/T	A/A	G/G	A/A	C/C
	5	T/T	A/A	A/A	A/A	T/T	A/A	G/G	A/A	C/C
NPRL-S122	1	T/T	C/C	G/G	C/C	C/C	A/G	G/G	G/G	C/C
	2	T/T	C/C	G/G	C/C	C/C	A/G	G/G	G/G	C/C
	3	T/T	C/C	G/G	C/C	C/C	A/G	G/G	G/G	C/C
	4	T/T	C/C	G/G	C/C	C/C	A/G	G/G	G/G	C/C
	5	T/T	C/C	G/G	C/C	C/C	A/G	G/G	G/G	C/C
NPRL-S118	1	T/T	C/C	A/A	A/A	T/T	A/A	A/A	A/A	A/A
	2	T/T	C/C	A/A	A/A	T/T	A/A	A/A	A/A	A/A
	3	T/T	C/C	A/A	A/A	T/T	A/A	A/A	A/A	A/A
	4	T/T	C/C	A/A	A/A	T/T	A/A	A/A	A/A	A/A
	5	T/T	C/C	A/A	A/A	T/T	A/A	A/A	A/A	A/A
I0322	1	T/T	C/C	A/A	A/A	T/T	A/A	A/A	A/A	A/A

**Note:**

SNP alleles are given in parentheses.

The SNP profile of the resistant line I0322 was identical to the five biological replicates of NPRL-S118 at the nine loci interrogated by the rhAmp assays. In a broader context, three of these loci (markers rh147, rh688, and rh194) are informative (genotype-specific) to discriminate accession NPRL-S118 from all runner type cultivars assessed in our comparative analysis (Fig. 1) and could potentially be used to confirm F_1_ hybrids.

The assays further uncovered genetic variation within accessions. Two biological replicates within accession NPRL-S120 (Table 2, biological replicates 2 and 3) were consistently different from the other three (biological replicates 1, 4 and 5) in 44% of the assays (rh621, rh294, rh506, rh688). These findings suggest the presence of at least two genotypes within accession NPRL-S120. Variation within accessions was also detected in NPRL-S121 with three biological replicates (Table 2, biological replicates 3, 4, and 5) showing unexpected genotypes at ≤ 22% of the SNP loci.

### Marker-trait association

**Figure 3:**
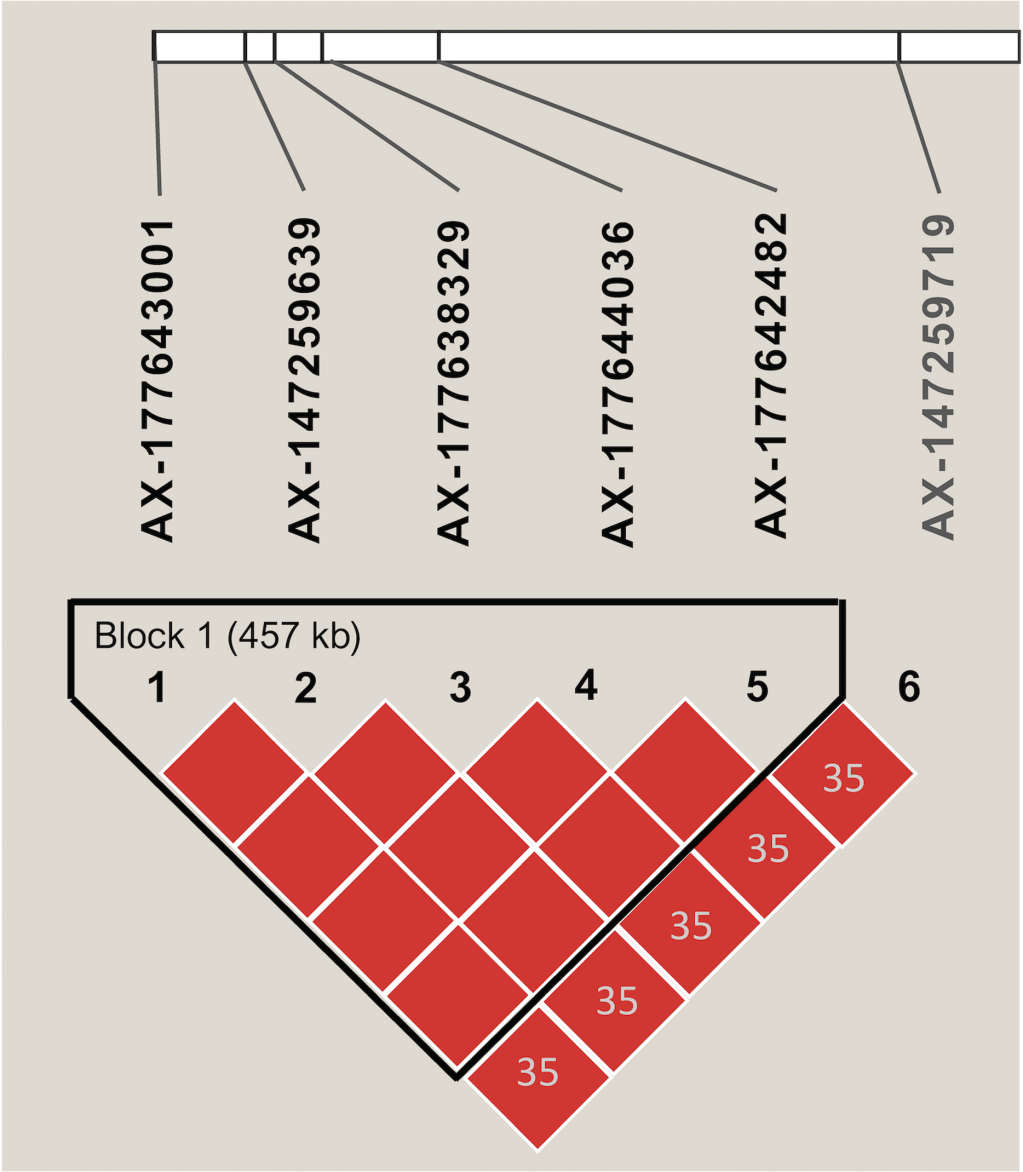
Linkage disequilibrium pattern of SNPs associated with disease severity index and disease incidence on chromosome Arahy.19. The graphic shows the haplotype block as defined by Haploview. Numbers within diamonds indicate the pairwise linkage disequilibrium values (*r^2^*) < 0.80 and red color within each block denotes *r*^2^ values equal or greater than 0.80. The length of each block is indicated in kilobases (kb).

Markers with significant segregation distortion (*P* < 0.01) accounted for less than 4% (138) of the 4,399 segregating SNPs. A final set of 4,261 markers were used in the association analysis, all of which were polymorphic between the two parental lines (Table S2). Allele contribution (%) from each parent is indicated in Fig. S3. Initial screening using simple linear regression detected 22 SNPs that were statistically significant (*P* < 0.05) at six independent regions of the A (Arahy.03, Arahy.05, Arahy.09) and B (Arahy.13, Arahy.18, and Arahy.19) sub-genomes (Table S7). The results from multiple regression further indicated that SNPs within each chromosome region were tagging the same variant, while only two independent regions (Arahy.09 and Arahy.19) remained significant. The final model with two SNPs, AX-147232560 and AX-177638329, which were selected to tag Arahy.09 and Arahy.19, respectively, explained the highest amount of phenotypic variance, with an adjusted *R*^2^ value of 0.66 (*P* < 0.001) for IN and 0.62 (*P* < 0.001) for DI. Alleles associated with resistance derived from the resistant parent I0322 (Fig. S4). The significant SNP AX-177638329 was in linkage disequilibrium (*r*^2^ > 0.80) with AX-177643001, AX-147259639, AX-177644036, and AX-177642482 (Fig. 3). This block spanned approximately 457 kb on chromosome Arahy.19 (from ~0.675 Mb to 1.121 Mb) of the *A. hypogaea* cv. Tifrunner reference genome (Fig. 3). On chromosome Arahy.09, AX-147232560 (6.9 Mb) was linearly correlated with AX-147232553 (7.3 Mb). Both genomic regions (Arahy.09, Arahy.19) bear genes known to be involved in defense responses to biotic stress, including leucine-rich repeat (LRR), zinc finger MYM-type, and WD repeat-containing protein families. On chromosome Arahy.09, in particular, the two SNPs are located within a disease-resistance hotspot containing several copies of TIR-NBS-LRR-encoding genes (https://peanutbase.org).

## Discussion

This study confirmed the presence of a germplasm in the U.S. peanut collection that is 98.6% identical (*P* < 0.01, bootstrap *t*-test) to the resistant line I0322 used by Bressano et al. (2019). Genetically, this is a very high level of identity for a germplasm that has been maintained separately, in different countries, for over 30 years. Similar values of identity have been reported for potato germplasm between the in vitro and the mother plants (Ellis et al., 2018), and identity values of ≤ 98.0% are expected between identical maize inbred lines maintained at different laboratories for long periods of time (Yan et al., 2009). While no inference can be made about shared resistant alleles between the resistant line I0322 and accession NPRL-S118 (PI 497287), the high level of similarity over a large number of loci distributed across the genome, and the occurrence of identical alleles surrounding significant SNPs, increase the probability that genes at causal loci are identical by descent (Meuwissen & Goddard, 2001).

The genetic basis of resistance to peanut smut has yet to be determined. Bressano et al. (2019) reported one SSR marker (cont01277a) associated with resistance in the JS3411 population, although not anchored to a genetic map. The current study detected SNPs markers mapped to chromosomes Arahy.09 and Arahy.19, suggesting at least two independent sources. However, our results of marker trait association will need to be validated in larger populations, due to the number of RILs used in this study. Given the small sample size, the analysis could be overestimating the magnitude of the observed genetic effect (e.g., *R*^2^) or failing to detect minor effects (Hong & Park, 2012). Nevertheless, disease resistance genes present in both genomic regions merit further attention. The region of chromosome Arahy.09 (~6.9–7.3 Mb), bearing the TIR-NBS-LRR-encoding genes, is of particular importance because it is also a source of root-knot nematode (*Meloidogyne arenaria* (Neal) Chitwood) resistance, originated from the wild A-genome species *Arachis cardenasii* Krapov. & W.C. Greg and introgressed into cultivated peanut by backcrossing (Bertioli et al., 2016; Burrow et al., 1996; Nagy et al., 2010).

The rhAmp technology used here for small-scale genotyping has advantage over previous PCR allelic discrimination technologies such as TaqMan and KASP by being almost five-fold more sensitive than KASP, four-fold less expensive than TaqMan, while resulting in better allele discrimination (Broccanello et al., 2018). In addition, rhAmp is particularly suitable for working in plant breeding given its flexibility about DNA quality and quantity (Broccanello et al., 2018). In the present study, nine rhAmp assays successfully validated SNP markers as well as genetic variation within accessions (i.e., NPRL-S120 and NPRL-S121). The latter emphasizes the need of an efficient and cost-effective genotyping system for detecting within-accession variation in the U.S. peanut germplasm collection. While genebanks seek to preserve the genetic composition of the original sample, in many cases, such as in peanut landraces, this involves maintaining genetically heterogenous accessions in a way that is difficult to use for gene discovery and crop breeding (Arias et al., 2018; Barkley et al., 2016; McCouch et al., 2012).

Landraces from Bolivia, as is the case of the PI accessions of *A. hypogaea* subsp. *fastigiata* used in this study, were collected decades ago from farm stores, local markets, and seed storages, many of which were obtained as a mixture of testa-color seeds (Krapovickas et al., 2009). Farmers in those areas often maintain this variability to guarantee a sustainable food supply to local communities; thus, such variability has been explained, in part, as the result of spontaneous cross-pollination by insects in the field (Krapovickas et al., 2009). The rate of cross-pollination in peanut is generally low (≤2%), however outcrossing can occur up to 8%, depending on genotype and year (Coffelt, 1989; Culp, Bailey & Hammons, 1968; Knauft, Chiyembekeza & Gorbet, 1992). From an evolutionary perspective, genetic exchange between sub-genomes (homoeologous recombination), deletions, and activity of mobile elements (transposable elements) have been documented and proposed as mechanisms contributing to the diversity of the allotetraploid *A. hypogaea* (Bertioli et al., 2019; Leal-Bertioli et al., 2018; Moretzsohn & Bertioli, 2018). Still, there is limited information about the genetic structure of accessions introduced from Bolivia.

Results from the present study provided insights into the genetic composition of the five PI accessions from Bolivia that share a common origin with a smut resistant line. In addition to NPRL-S118, three other accessions were closely related to the resistant line I0322 and merit further assessment. All five PI accessions from the collection have been classified as *A. hypogaea* L. subsp. *fastigiata* var. *fastigiata* (NPGS), however, we speculated that accession NPRL-S122 belongs to the *A. hypogaea* L. subsp. *hypogaea* var. *peruviana* described for Bolivia (Krapovickas et al., 2009). Genetic relationships among 20 peanut genotypes from a peanut diversity panel placed accession NPRL-S122 closer to a genotype of *A. hypogaea* L. subsp. *hypogaea* var. *hirsuta* Köhler than to accessions of subspecies *fastigiata* var. *fastigiata* collected from Bolivia (Fig. 3). This is consistent with previous studies showing that var. *peruviana*, which is likely the case of NPRL_222, is closer to subspecies *hypogaea* than to subspecies *fastigiata* (Cuc et al., 2008; Freitas, Moretzsohn & Valls, 2007; He & Prakash, 2001; Raina et al., 2001). Nevertheless, more investigation is required to elucidate the genetic identity of accession NPRL-S122.

## Conclusions

This study implemented an efficient and cost-effective genotyping approach to verify whether the source of a highly resistant (nearly immune) germplasm line (I0322; Bressano et al., 2019) was present in the U.S. peanut germplasm collection. By combining large-scale and small-scale genotyping, we were able to trace the I0322 resistant line back to accessions of *A. hypogaea* subspecies *fastigiata* introduced into the U.S. more than 30 years ago. The results provided statistically significant evidence that the U.S. peanut collection contains at least one putative smut-resistant germplasm line. The marker-trait association analysis based on the Bressano et al. (2019) RIL population, albeit limited in number, suggested two independent sources of resistance. Nevertheless, these results may not be applicable to the PI lines evaluated in this study unless one of them is indeed identical or nearly identical by descent.

## Supplemental Information

10.7717/peerj.10581/supp-1 Supplemental Information 1 Neighbor-joining tree based on pairwise genetic distance among 11 genotypes, using 14,298 SNPs. Scale bar at the bottom of the dendrogram indicates the proportion of loci for which individuals differ. Scale bar below each seed and pod image corresponds to 1 cm.

10.7717/peerj.10581/supp-2 Supplemental Information 2 SNP frequency distribution across the 20 peanut chromosomes. Frequency is expressed in number of occurrences per 100 kb. The number of SNP differences between each of the PI accessions and the resistant line I0322 were plotted against the physical position of the chromosome.

10.7717/peerj.10581/supp-3 Supplemental Information 3 Percentage of parent-specific alleles contributed to each of the progeny in the cross JS31411. The plot is based on 4,261 SNPs, which were polymorphic between the parental lines.

10.7717/peerj.10581/supp-4 Supplemental Information 4 Box plots showing the effects of the marker genotypic classes (0,2) of two candidate SNPs for disease incidence (IN). Asterisks indicate the SNP allele from the resistant parent (I0322).

10.7717/peerj.10581/supp-5 Supplemental Information 5 Summary of rhAmp genotyping assays with SNP IDs, chromosome (Chr), SNP flanking sequences, and RNase H-dependent PCR primers used for rhAmp SNP genotyping. Allele primers: numbers 1 and 2; locus specific primers: LS.

10.7717/peerj.10581/supp-6 Supplemental Information 6 Genotype calls of 16 RILs derived from the cross I0322 × Guasu (JS31411) with smut disease incidence (%) and disease index mean values.

10.7717/peerj.10581/supp-7 Supplemental Information 7 Genotype calls of the five PI accessions and the resistant line I0322 used in this study, based on 14,298 SNP markers of the Axiom Arachis2 SNP array. AA:0, BB:2, No call:-1. SNPs related to Figure 1 and Table S5 are indicated with asterisk.

10.7717/peerj.10581/supp-8 Supplemental Information 8 Distribution of 14,298 SNPs across the 20 *Arachis* chromosomes, which is based on the *A. hypogaea* cv. Tifrunner reference genome.

10.7717/peerj.10581/supp-9 Supplemental Information 9 Cluster of SNPs called as heterozygous on chromosome group 4, with corresponding chromosome location and target gene/intergenic region. SNP location is based on both, the *A. hypogaea* cv. Tifrunner reference genome and Axiom Arachis2 SNP array.

10.7717/peerj.10581/supp-10 Supplemental Information 10 Pairwise genetic distance matrix of 26 genotypes and 4,871 SNPs. The underlined names are the 20 genotypes extracted from Clevenger et al. (2018).

10.7717/peerj.10581/supp-11 Supplemental Information 11 Statistically significant SNPs (*P* < 0.05) detected after the initial screening using simple linear regression. SNP physical location is based on the *A. hypogaea* cv. Tifrunner reference genome.

## Additional Information and Declarations

### Data Availability

The following information was supplied regarding data availability: The raw genotype data are available in the Supplemental Files. The Plant Introduction (PI) accession numbers are: PI 497283-87.
