# Supplementary figures and images for: Genotyping tools and resources to assess peanut germplasm: smut-resistant landraces as a case study
Source: PeerJ. 2021 Jan 29;9:e10581. doi: 10.7717/peerj.10581 (PMC7849506; doi:10.7717/peerj.10581)

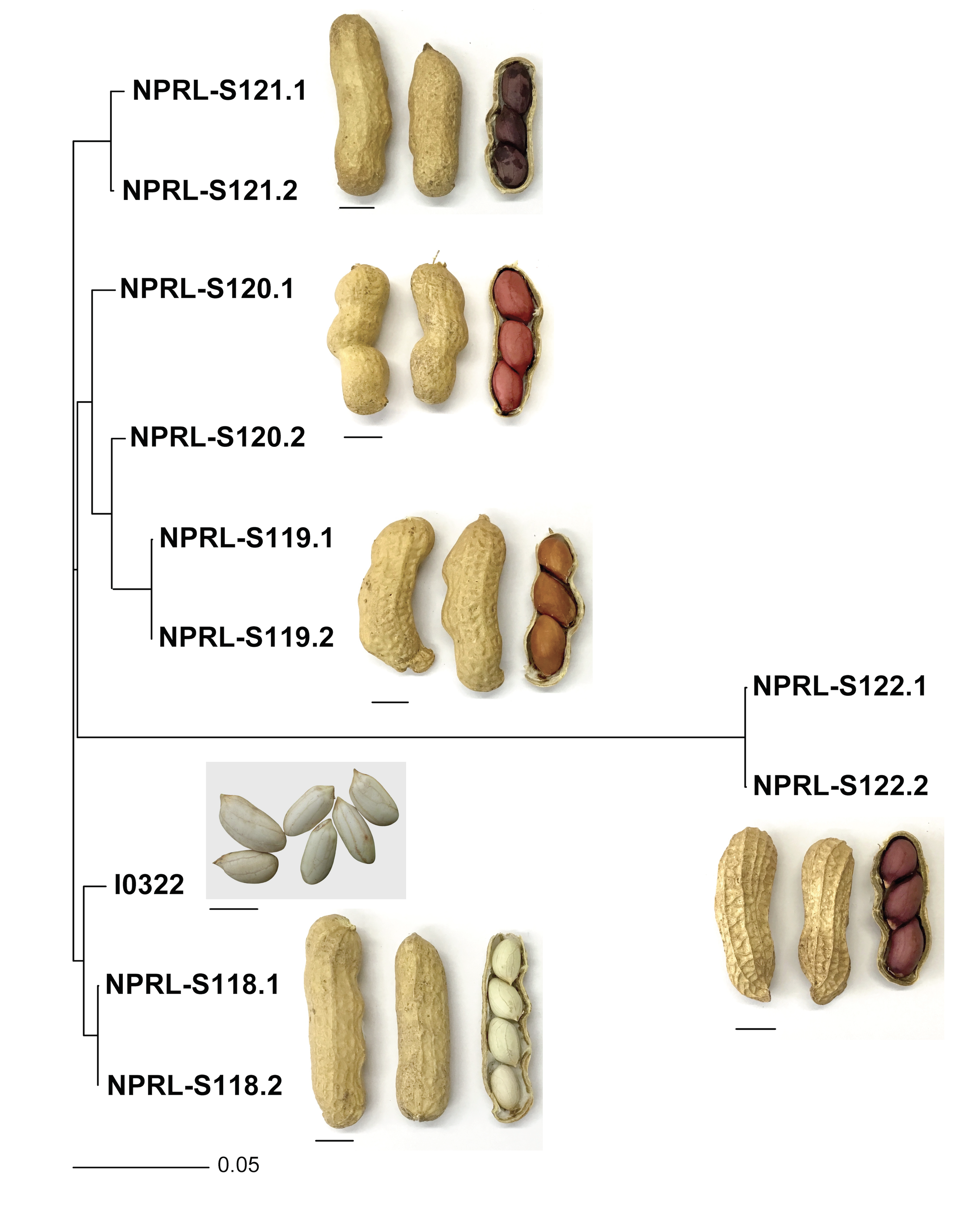

Supplement: Supplemental Information 1 — Scale bar at the bottom of the dendrogram indicates the proportion of loci for which individuals differ. Scale bar below each seed and pod image corresponds to 1 cm. [file peerj-09-10581-s001.png]

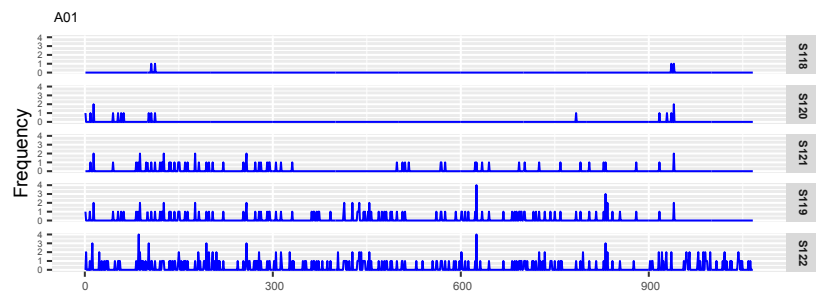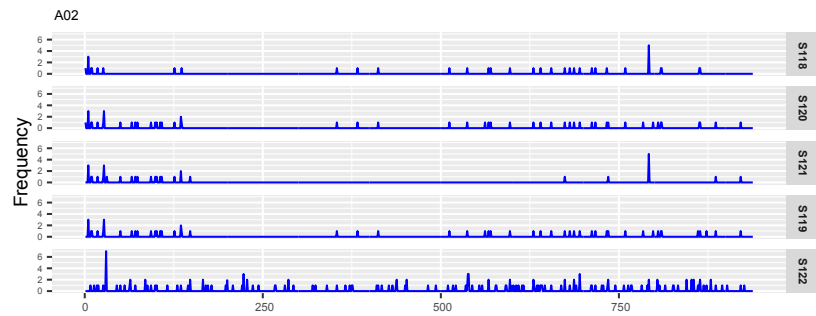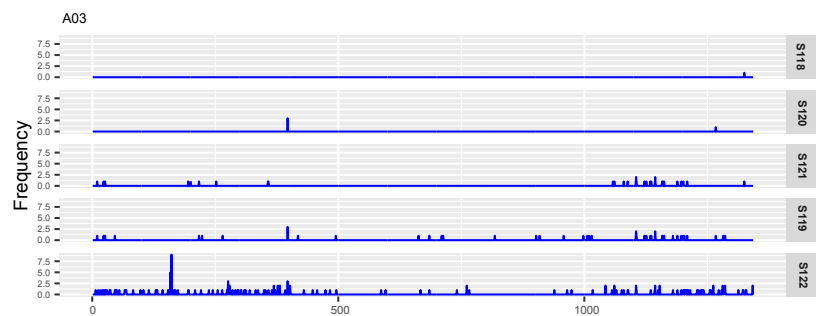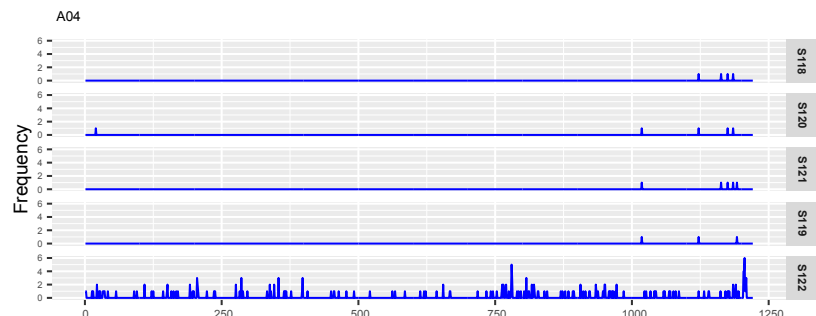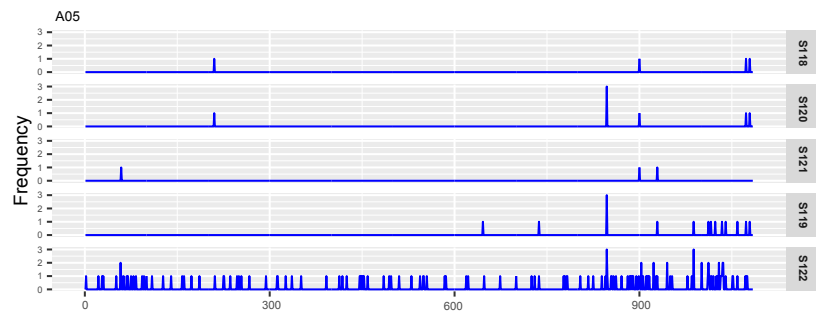

SNP position (100Kb bins)

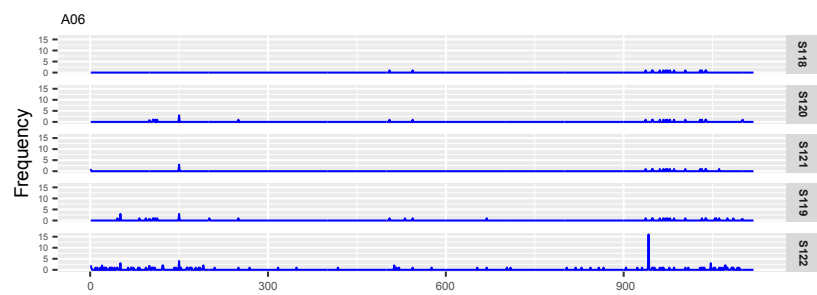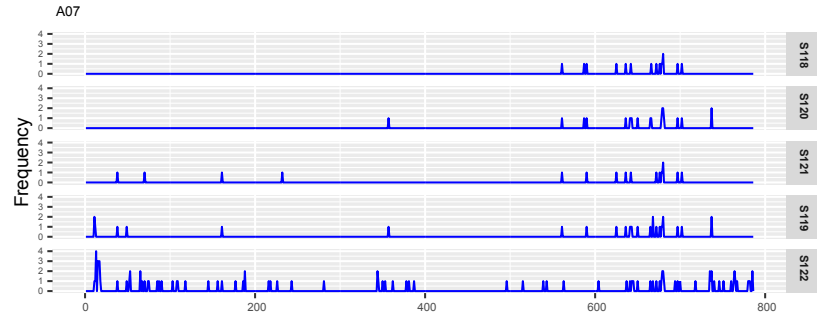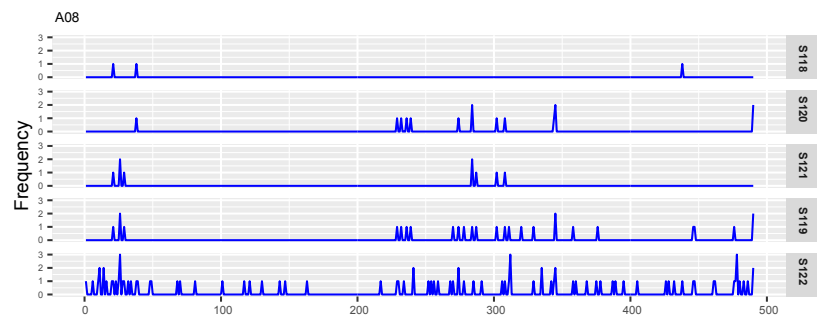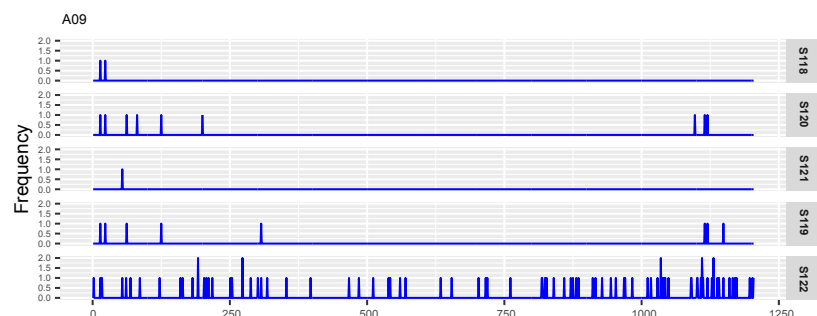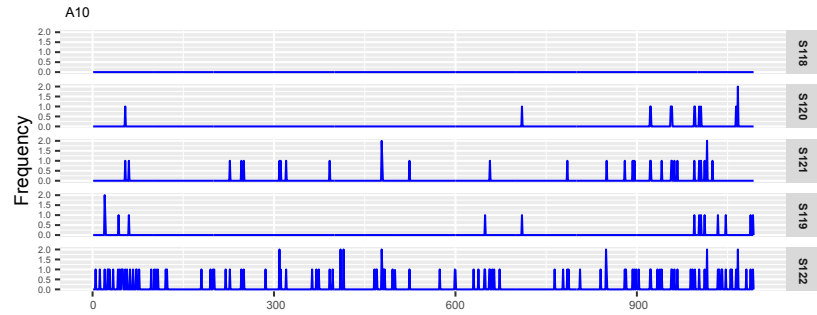

SNP position (100Kb bins)

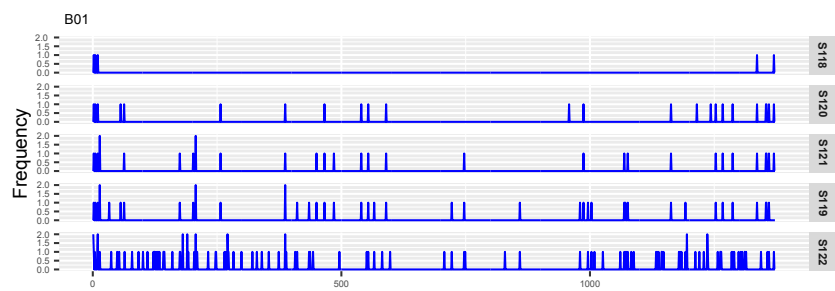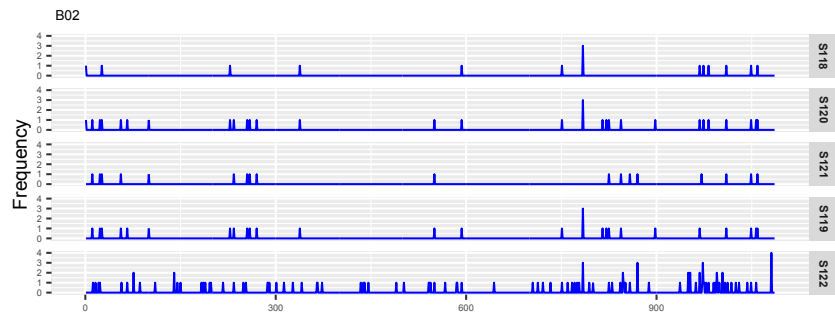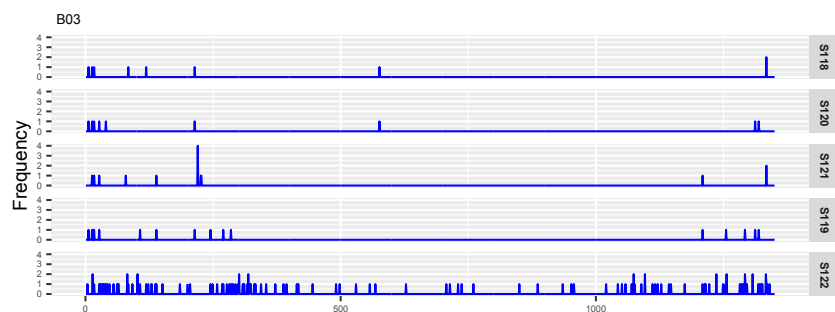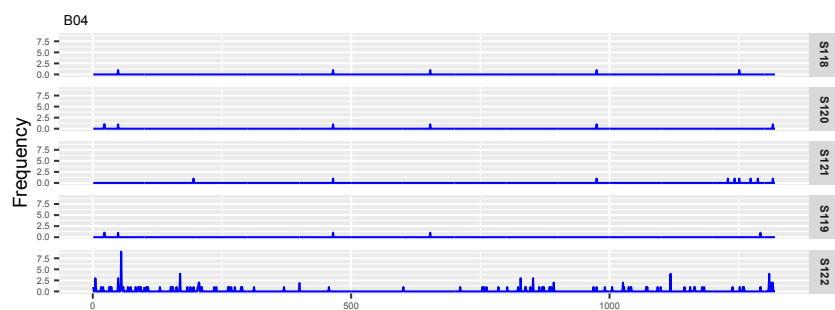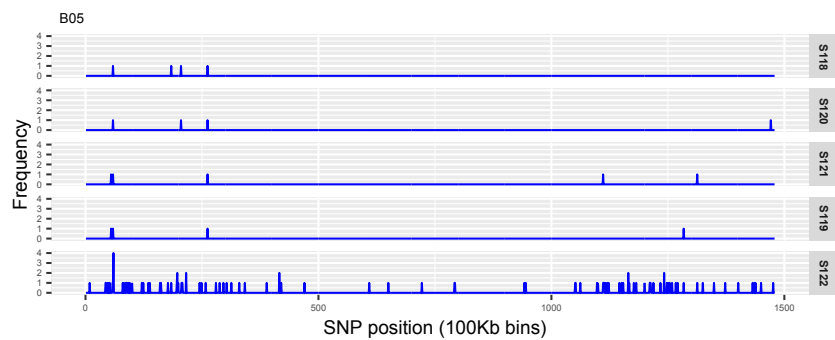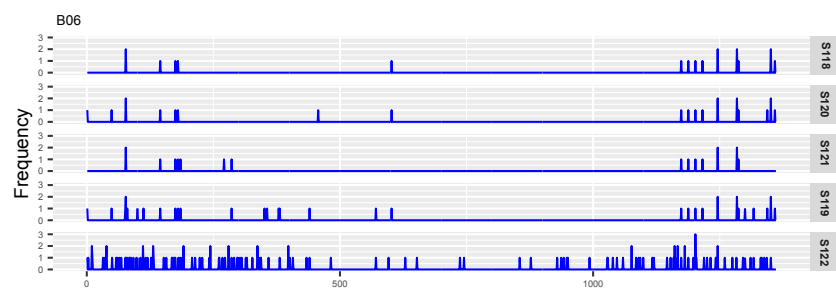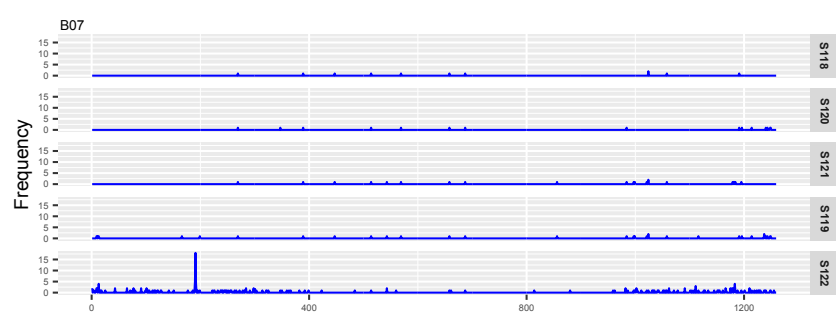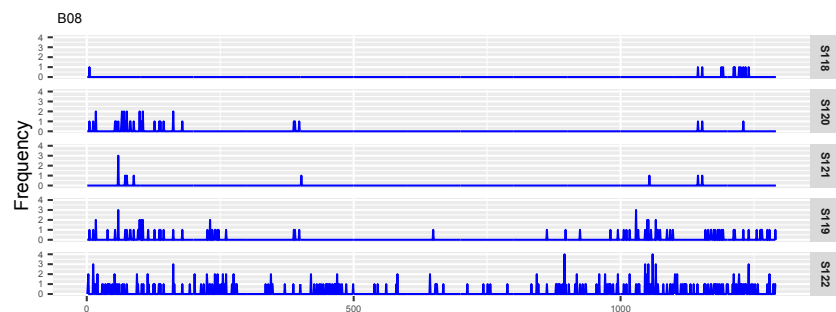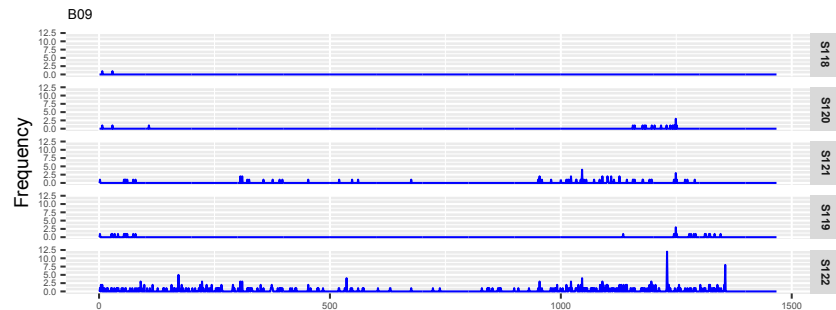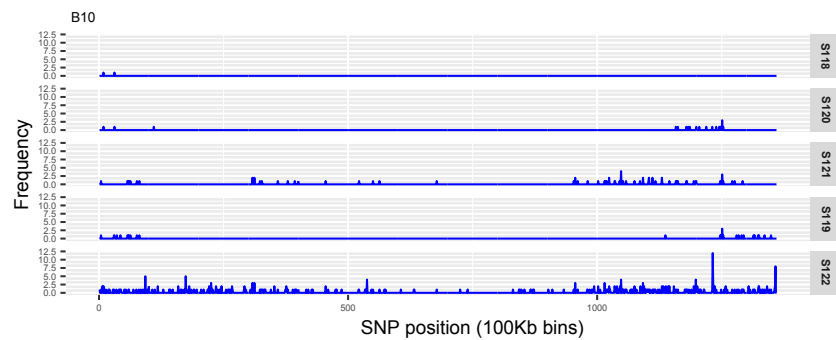

Supplement: Supplemental Information 2 — The number of SNP differences between each of the PI accessions and the resistant line I0322 were plotted against the physical position of the chromosome. [file peerj-09-10581-s002.pdf]

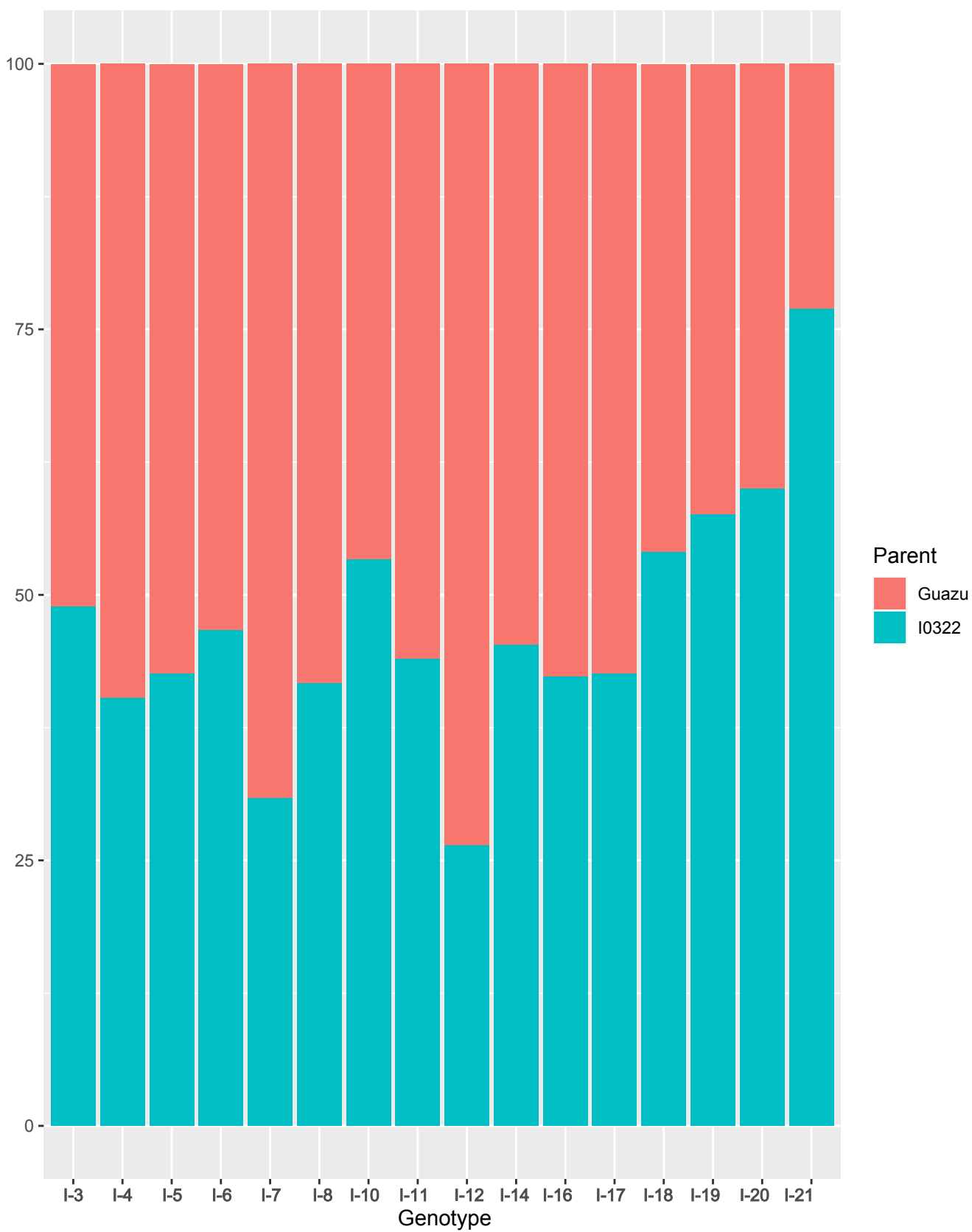

Supplement: Supplemental Information 3 — The plot is based on 4,261 SNPs, which were polymorphic between the parental lines. [file peerj-09-10581-s003.pdf]

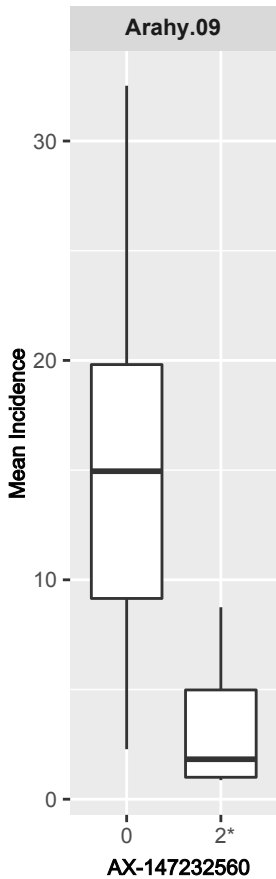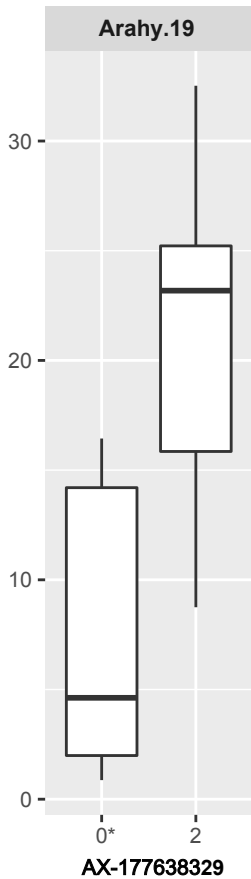

Supplement: Supplemental Information 4 — Asterisks indicate the SNP allele from the resistant parent (I0322). [file peerj-09-10581-s004.pdf]
