# Supplementary material for: Genotyping tools and resources to assess peanut germplasm: smut-resistant landraces as a case study
Source: PeerJ. 2021 Jan 29;9:e10581. doi: 10.7717/peerj.10581 (PMC7849506; doi:10.7717/peerj.10581)
Supplement: Supplemental Information 5 — Allele primers: numbers 1 and 2; locus specific primers: LS. [file peerj-09-10581-s005.docx]

| Axiom Arachis2 SNP ID | Chr | SNP flanking sequences with evaluated SNPs (indicated by brackets) submitted to the rhAmp Genotyping Design Tool | rhAmp assay | Primer | Sequence 5' → 3 |
| --- | --- | --- | --- | --- | --- |
| AX-147232621 | Arahy.09 | TAGTTTTATTCCTACAGGACTTCTATTCTCCATCTAGGGTTTCTTTCTCCGTCATCTCCA[C/T]TGACACTCGAACCCAGAGATGTCCTTTACAGCTAAAGATCATGGCACGATCAAACTTTAA | rh621 | 1  2  LS | TCTGGGTTCGAGGTCAG  TCTGGGTTCGAGTGTCAA  GCTCCTACAGGACTTCTATTCTCCAT |
| AX-147241664 | Arahy.12 | ACCAATCAGGGATATCAGTTGTACCAACCATCAATGCAAGGTTACAGACTGGATTTCTGG[A/C]AGCTGCAGCAATAAACTTATCTGGTGCCTAAGCAATCACACACATATGATCAAACTCTGA | rh664 | 1  2  LS | GTTACAGACTGGATTTCTGGA  TTACAGAGTGGATTTCTGGC  GCTCAGAGTTTGATCATATGTGTGTG |
| AX-176792019 | Arahy.06 | TGTTGTTCATGGCGGGCACCACATACTGCGCATAAATTTCATTTGGTTTGACTTC[A/G]TTTCCAACGGAGATGTATCTGAACCTCACATTTCCATTTCCATAGTTTCTTACGT | rh019 | 1  2  LS | TAGATACATCTCCGTTGGAAAT  TCAGATACATCTCCGTTGGAAAC  GCCACCACATACTGCGCATAAA |
| AX-177640147 | Arahy.07 | CAGTTACAGTGTGGTGGTGTCTTCTGAAAGCTTCGGGGAACCAAAAGTTTTGATT[A/C]AGTGAAGGTGCAGGTTTAGGTGCATGTGTGGATCCCATTCCCTTCCGCGTTAATG | rh147 | 1  2  LS | CTAAACCTGCACCTTCACTT  CTAAACCTGCACCTTCACTG  GCTACAGTGTGGTGGTGTCTT |
| AX-176792294 | Arahy.05 | CTCATGGACAAAAGTGAGAAACTTATTATAATTATTTTACAATTTGACCGAA[C/T]AACTGCCAAAGCATCAAATCAATGATTTTGTAATTCTCCTTCTGATGGTTGAT | rh294 | 1  2  LS | TTATAATTATTTTACAATTTGACCGAAC  TTATAATTATTTTACAATTTGACCGAAT  GCTCAACCATCAGAAGGAGAATT |
| AX-147226921 | Arahy.07 | AAAAGATACTTTTAATTAGTACGAGCGATATCTGAATGGTTGAAACAAGT[A/G]CATAGTTTTCTATAAGGAACATCTTTCAGCCAAGCAAAATAAGTGTCTTGGGA | rh921 | 1  2  LS | TGAAAGATGTTCCTTATAGAAAACTATGT  TGAAAGATGTTCCTTATAGAAAACTATGC  GCATTAGTACGAGCGATATCTGAATG |
| AX-176818506 | Arahy.03 | GAGGAAGGAAAGTTAATGAGAGAGAAAGCGGTGTTGATGAAGGATAAAATTAAGATAAGT[A/G]TTAGAGATAATGATGCTTTAAATGGAATTGTTCATGCTATCGTCGGGTCTTCTTCTAATT | rh506 | 1  2  LS | ATTCCATTT AAAGCATCATTATCTCTAAT  ATTCCATTT AAAGCATCATTATCTCTAAC  GCAGCGGTGTTGATGAAGGATA |
| AX-177643688 | Arahy.10 | TCTTCCCAGTGTCCAGCTCTAGCAGGGAACTCACCCATCGGCGGCAACAGATGCAACAAT[A/G]GCGACGAGTTATAACAGCTCGACGATGGCGAGGCAGAACGGCAGCAATCTTTCTTCCATG | rh688 | 1  2  LS | GAGCTGTTATAACTCGTCGCT  GAGCTGTTATAACTCGTCGCC  GCTCCAGCTCTAGCAGGGA |
| AX-176795194 | Arahy.01 | ACGGAGTCGACGGTAGTAAGACTTAAATAAATGTAGAAATGTTGAAAAGGGTGAAGTGGG[A/C]GTCGGTGGCAAGTAATGACACGTGCCAGTGGCACACAACCTTTGGGTGATTGCCAATTTG | rh194 | 1  2  LS | TCATTACTTGCCACCGACT  TCATTACTTGCCACCGACG  GCACGGAGTCGACGGTAGT |
